# Responsiveness of prepubertal crossbred bull calves to exogenous GnRH and its impact on reproductive hormones under tropical conditions

**DOI:** 10.1186/s40064-016-1956-4

**Published:** 2016-03-08

**Authors:** B. S. Bharath Kumar, Sujata Pandita, B. S. Prakash, Smrutirekha Mallick, Bhabesh Mili

**Affiliations:** Dairy Cattle Physiology Division, Lab No - 216, ICAR-National Dairy Research Institute, Karnal, Haryana 132001 India; Department of Animal Physiology and Nutrition, Indian Council of Agricultural Science, Pusa, New Delhi, India

**Keywords:** Androstenedione, Gonadotropin-releasing hormone, Luteinizing hormone, Testosterone, Total estrogens

## Abstract

This study investigated the age related variations in luteinizing hormone (LH), androstenedione, testosterone, and total estrogens response to exogenous gonadotropin-releasing hormone (GnRH) in Holstein–Friesian (HF) × Tharparkar bull calves. Fifteen bull calves were selected and, based on their age, were divided into Group I (14–16 months, n = 5), Group II (9–12 months, n = 5), and Group III (6–8 months, n = 5). All bull calves were administered with 10 µg of GnRH intramuscularly. Blood samples were collected at an interval of 30 min commencing 1 h prior to GnRH treatment until 4 h post-GnRH treatment and thereafter, at an interval of 1 h for the next 3 h. Endocrine response in terms of pretreatment values, peak values, area under curve, and time taken to attain peak values for LH, androstenedione, testosterone, and total estrogens was evaluated in all the bull calves. Significant differences were observed in pretreatment values, peak concentrations, and area under curve for androstenedione and testosterone between the groups; with response being higher in Group I bull calves. The results indicated that the HF × Tharparkar bull calves of 14 months age and above respond to exogenous GnRH by secreting significant amounts of testosterone.

## Background

The Holstein–Friesian (HF) × Tharparkar has evolved as a potent milk producer among various cross breeds in Tropical India. However, male offspring’s of this cross breed are inferior to *Bos indicus* and *Bos taurus* in semen production. Impaired semen production capacity, poor libido, and low freezability are the major reasons for rejection of these crossbreds in semen stations (Sethi et al. 1989; Bhavsar 1993; Sahni and Mohan 1998). From the data collected over a period of 15 years, Mukhopadhyay et al. (2010) observed that the mean ± SE age at first semen collection (AFSC) in HF × Tharparkar bulls was 872 ± 19.1 days (~27 months). Testosterone and gonadotropins are essential to initiate and support the process of spermatogenesis (Kerr et al. 1993). Follicle stimulating hormone acts synergistically with testosterone to influence the efficiency of spermatogenesis and fertility (Sharpe 1994; Mc Lachlan et al. 1994). The trend line for testosterone profiles in growing Sahiwal (indigenous breed) males indicated an exponential increase in testosterone with age when compared to an almost linear increase in HF × Tharparkar males (Gulia et al. 2010), indicating low testosterone production with age as probable cause for poor libido and poor semen production in these crossbred males.

Administration of GnRH analogue, on a weekly basis, to Egyptian puberal buffalo bulls of 15–18 months of age significantly improved the libido and semen quality (El-Khawaga et al. 2011). Bulls provided with additional energy in the diet combined with weekly administration of GnRH significantly increased the testosterone levels and scrotal circumference in comparison to bulls that were fed only with the additional energy in the diet (Ali et al. 2012). The combined strategy of providing additional energy in the diet with weekly administration of GnRH to pre-pubertal HF × Tharparkar bull calves might augment testosterone levels and decrease the AFSC. For these reasons, the age at which the pre-pubertal bull calves respond to exogenous GnRH by secreting significant amounts of testosterone has to be investigated. Hence, this study was designed with an objective to determine the appropriate age at which pre-pubertal HF × Tharparkar bull calves are responsive to exogenous GnRH.

## Results

**Fig. 1:**
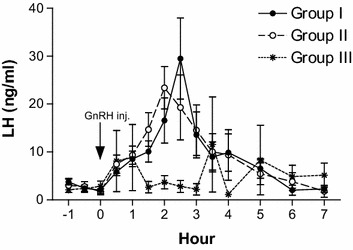
Plasma LH profile of Group I (14–16 months, n = 5), Group II (9–12 months, n = 5), and Group III (6–8 months, n = 5) Holstein × Tharparkar bull calves, before and after GnRH administration

Comparison of mean pre-treatment and peak concentrations of hormones among the groups are given in Tables 1 and 2, respectively. The mean pre-treatment LH levels were as low as 3.75 ± 0.61, 3.02 ± 1.46, and 2.12 ± 0.49 ng/ml in Group I, Group II, and Group III bull calves, respectively. The pattern of LH release before and after GnRH administration in Group I, Group II, and Group III bull calves is shown in Fig. 1. Significant increase in LH after GnRH administration was observed in all groups. Among the bull calves of Group I, the LH levels rose gradually after GnRH administration and reached a peak, which ranged between 14.3–59.6 ng/ml. The peak LH levels in all the bull calves of Group I were observed at 2½ h post GnRH administration. Similar gradual increase in LH concentrations was observed in Group II bull calves and the peak levels ranged between 14.3–43.9 ng/ml. The peak LH levels among Group II bull calves were observed either at 2 or 2½ h post GnRH administration. There was no particular pattern of LH release among Group III bull calves and large fluctuation was observed in the duration at which the peak levels were attained.

**Table 1:** Mean ± SE pre-treatment concentrations of LH, androstenedione, testosterone, and total estrogens in Group I (14–16 months), Group II (9–12 months), and Group III (6–8 months) Holstein × Tharparkar bull calves

Hormones	Group I	Group II	Group III
LH (ng/ml)	2.90 ± 0.84^a^	2.35 ± 0.67^a^	2.46 ± 0.35^a^
Androstenedione (ng/ml)	2.10 ± 0.17^a^	0.79 ± 0.06^b^	0.05 ± 0.01^c^
Testosterone (ng/ml)	0.97 ± 0.08^a^	0.32 ± 0.06^b^	0.19 ± 0.03^b^
Total estrogens (pg/ml)	43.1 ± 23.6^a^	47.3 ± 26.0^a^	20.93 ± 5.48^a^

^a, b^Means of pre-treatment values within rows without a common letter differ significantly by one-way of variance (ANOVA)

**Table 2:** Mean ± SE peak concentrations of LH, androstenedione, testosterone, and total estrogens in Group I (14–16 months), Group II (9–12 months), and Group III (6–8 months) Holstein × Tharparkar bull calves

Hormones	Group I	Group II	Group III
LH (ng/ml)	29.5 ± 8.49^a^	23.4 ± 4.41^a^	11.6 ± 9.91^a^
Androstenedione (ng/ml)	3.01 ± 0.72^a^	2.59 ± 0.71^a^	0.31 ± 0.22^b^
Testosterone (ng/ml)	3.04 ± 0.29^a^	0.93 ± 0.27^b^	0.52 ± 0.17^b^
Total estrogens (pg/ml)	21.8 ± 4.7^a^	29.9 ± 14.4^a^	26.0 ± 11.0^a^

^a, b^Means of peak values within rows without a common letter differ significantly by one-way of variance (ANOVA)

**Fig. 2:**
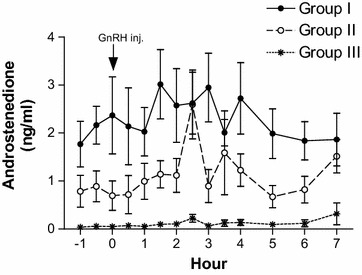
Plasma androstenedione profile of Group I (14–16 months, n = 5), Group II (9–12 months, n = 5), and Group III (6–8 months, n = 5) Holstein × Tharparkar bull calves, before and after GnRH administration

**Fig. 3:**
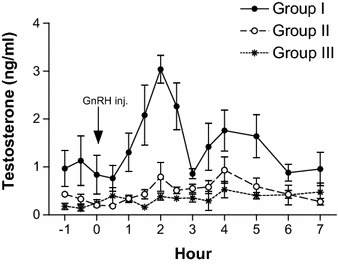
Plasma testosterone profile of Group I (14–16 months, n = 5), Group II (9–12 months, n = 5), and Group III (6–8 months, n = 5) Holstein × Tharparkar bull calves, before and after GnRH administration

The androstenedione (Fig. 2) and testosterone (Fig. 3) response to exogenous GnRH exhibited a significant rise with age. The pre-treatment androstenedione concentrations differed (*p* < 0.05) among all groups, whereas peak values differed (*p* < 0.05) only between group I and group III. Although, the mean androstenedione levels rose as high as 2.59 ± 0.72 ng/ml in Group II bull calves, the peak testosterone levels observed was around 1 ng/ml. Significant increase in testosterone after GnRH administration was observed only in Group I bull calves. The peak testosterone levels after GnRH administration among Group I bull calves ranged between 2.78 and 4.21 ng/ml, three out of five bull calves attained their testosterone peaks at 2 h post GnRH administration while the remaining two bull calves attained the peak levels at 2½ h post GnRH administration. The pre-treatment testosterone concentrations (*p* < 0.001) and peak values (*p* < 0.0001) were higher in the Group I bull calves than in the other groups. Both the androstenedione and testosterone concentrations in Group III bull calves remained below 1 ng/ml and the pre-treatment and peak level did not differ (*p* > 0.05).

**Fig. 4:**
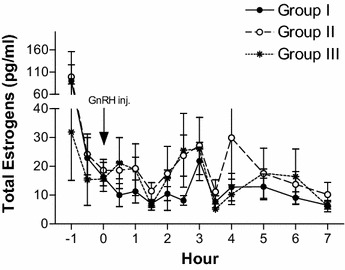
Plasma total estrogens profile of Group I (14–16 months, n = 5), Group II (9–12 months, n = 5), and Group III (6–8 months, n = 5) Holstein × Tharparkar bull calves, before and after GnRH administration

The mean areas under response curve of LH, androstenedione, testosterone, and total estrogens in Group I, Group II, and Group III bull calves are given in Table 3. The mean areas under the response curve for androstenedione differed (*p* < 0.01) between Groups I and III. The mean area under response curve for testosterone differed (*p* < 0.01) between Group I and Group II; and between Group I and Group III. The highest values of area under response curve among Group I, Group II, and Group III bull calves were 18.7, 5.39, and 4.90 ng/ml h, respectively; and the lowest values of area under response curve among Group I, Group II, and Group III bull calves were 6.01, 2.48, and 1.27 ng/ml h, respectively. The mean pre-treatment, peak, and area under response curve values did not differ (*p* > 0.05) for total estrogens between the groups. However, higher pre-treatment concentrations of total estrogens were observed in all the groups and the concentrations decreased after the administration of GnRH (Fig. 4).

**Table 3:** Mean ± SE area under response curve of LH, androstenedione, testosterone, and total estrogens in Group I (14–16 months), Group II (9–12 months), and Group III (6–8 months) Holstein × Tharparkar bull calves

Hormones	Group I	Group II	Group III
LH (ng/ml h)	67.7 ± 16.4^a^	69.4 ± 15.9^a^	39.7 ± 19.4^a^
Androstenedione (ng/ml h)	18.2 ± 4.32^a^	8.32 ± 2.93^a, b^	0.91 ± 0.35^b, c^
Testosterone (ng/ml h)	11.4 ± 2.22^a^	3.63 ± 0.53^b^	2.8 ± 0.59^b, c^
Total estrogens (pg/ml h)	115 ± 29.8^a^	169 ± 22.5^a^	128 ± 44.9^a^

^a, b, c^Means within rows without a common letter differ significantly by one-way of variance (ANOVA)

## Discussion

To the best of our knowledge, this is the first report that has evaluated the appropriate age at which the pre-pubertal HF × Tharparkar bull calves are responsive to the exogenous GnRH under tropical environmental conditions. The non-significance in the LH levels among the different age groups after GnRH administration in the present study is similar to the results obtained by Mongkonpunya et al. (1975). The LH concentrations after GnRH administration in Hereford bulls as observed by Schanbacher and Echternkamp (1978) are in accord with our results. The mean peak concentration of testosterone in Group I bull calves was observed as early as 2 h, whereas in both Group II and Group III bull calves, the peak was observed at 4 h after GnRH administration (Fig. 3). The pre-treatment testosterone concentrations and their increment with age are in accord with the study conducted by Gulia et al. (2010) on HF × Tharparkar bulls and bull calves. The difference between pre-treatment and peak levels of androstenedione in Group II bull calves were significant (*p* < 0.05) while the difference between pre-treatment and peak testosterone levels did not differ (*p* > 0.05). Among the three groups, only Group I bull calves showed a significant (*p* < 0.0001) difference between the pre-treatment levels and the peak concentration of testosterone after GnRH administration, indicating the responsiveness to GnRH. Since testosterone is essential for initiation and maintenance of spermatogenesis (Kerr et al. 1993), the administered GnRH seemed to have a functional role only among Group I bull calves, i.e. bull calves of 14 months of age and above. The results suggest that the weekly administration of GnRH (Ali et al. 2012) in order to augment testosterone levels and decrease AFSC might be successful only in pre-pubertal HF × Tharparkar bull calves of 14 months of age and above.

Since the differentiation of Leydig cells is completed by 6 months of age in bull calves (Amann et al. 1986), steroidogenesis has to take place in presence of LH. In the present study, LH levels were increased in the bull calves of all the three age groups but significant (*p* < 0.0001) amounts of testosterone was produced only in Group I (14–16 months of age) bull calves whereas Mongkonpunya et al. (1975) observed testosterone levels as high as 5.30 ng/ml in 6 months old HF bull calves after administration of exogenous GnRH. Growth hormone, insulin like growth factor-1 (Lin et al. 1986; Horikawa et al. 1989), insulin-like peptide 3 (Pathirana et al. 2012) and other growth factors are required, in adequate amounts, for steroidogenesis and production of testosterone. Holstein–Friesian × Tharparkar crossbreds have not adapted to the tropical Indian environmental conditions (Gulia et al. 2010) and their body weight gain is poorer than the HF and Sahiwal breeds. The concentration of the metabolic hormones and growth factors vary with the body weight and growth (Verde and Trenkle 1987); this may be the reason for significant differences in the pre-treatment and post-treatment concentrations of testosterone between the bull calves of three different age groups in the present study and for lower magnitudes than the HF bull calves as observed by Mongkonpunya et al. (1975). The total estrogen levels did not differ after administration of exogenous GnRH in all the groups, which agreed with a similar study carried out on adult HF × Tharparkar bulls (Bharath Kumar et al. 2015). We did not find any significant difference in the peak concentrations and area under response curve of total estrogens and time taken to attain peak levels between the groups.

## Conclusions

From the present study, it was concluded that the HF × Tharparkar bull calves of 14 months age and above respond to exogenous GnRH by secreting significant (*p* < 0.0001) amounts of testosterone and the studies pertaining to decreasing the AFSC by frequent exogenous GnRH administration could be conducted on HF × Tharparkar bull calves of ≥14 months of age.

## Methods

### Selection, feeding, and management of bull calves

The experiment was conducted at National Dairy Research Institute (NDRI), Karnal, Haryana, India. Fifteen HF × Tharparkar bull calves were selected, and based on their age, were divided into group I (14–16 months, n = 5), group II (9–12 months, n = 5), and group III (6–8 months, n = 5). The mean ± SE body weights of group I, II, and III bull calves were 186.6 ± 5.16, 141.4 ± 3.31, and 104.6 ± 1.57 kg, respectively. The trial was conducted during the month of September. All the animals were housed in individual pens and were fed with maintenance ration as per kearl (1982) standards to gain @ 500 g/day. The concentrate offered consisted of 22 % CP and 70 % TDN and the concentrate mixture composed of 32 % groundnut cake, 33 % maize, 26 % wheat bran, 6 % de-oiled rice bran, 2 % mineral mixture, and 1 % salt. Concentrate was offered in the forenoon whereas adlib green fodder was offered in the afternoon. Water was offered twice daily. Approval for animal experimentation was obtained from the Institutional Animal Ethical Committee of National Dairy Research Institute (1705/GO/ac/13/CPCSEA Dt. 3/7/2013).

### Animal treatment, blood sampling, and hormone analysis

All the bull calves were treated with 10 µg of GnRH [Receptal^®^ (Buserelin acetate)] intramuscularly. The blood samples were collected at an interval of 30 min commencing 1 h prior to GnRH treatment until 4 h post-GnRH treatment and thereafter, at an interval of 1 h for the next 3 h. In all the bull calves, blood sampling and GnRH administration were carried out between 0700 and 1500 h. Immediately after collection, the blood samples were centrifuged at 1077×*g* for 15 min, and the plasma samples were stored at −20 °C until they were analyzed for testosterone (Gulia et al. 2010), luteinizing hormone (LH; Prakash et al. 2002), androstenedione (Mallick et al. 2015), and total estrogens (Mondal et al. 2006) by enzyme immunoassay procedures previously developed in the laboratory. The intra-assay coefficients of variation for testosterone, androstenedione, LH, and total estrogens were 3.12, 8.1, 6.48, and 2.97 %, respectively. The inter-assay coefficients of variation for testosterone, androstenedione, LH, and total estrogens were 7.43, 9.34, 14.21, and 9.68 %, respectively.

### Definition of pre-treatment concentration and statistical analysis

The average concentrations from the samples collected before and during the GnRH administration constituted the pre-treatment levels of the corresponding hormones. Repeated measures ANOVA was used to determine the significant increase in peak levels from the pre-treatment concentrations in each group for all hormones. Tukey’s multiple comparison tests (one way ANOVA) was used to compare the pre-treatment levels, peak concentration of hormones, and area under curve between Group I, Group II, and Group III bull calves. Student’s *t* test was employed to compare the pre-treatment concentrations with the peak levels of the hormones. The total area formed on graph between ½ h after GnRH administration till 7 h was considered for calculating area under curve for all the hormones. Graphpad prism (version 5) and SPSS (version 16) softwares were used for statistical analysis.

## References

[CR1] Ali S, Ullah N, Akhter S (2012). GnRH and diet effects testosterone and scrotal circumference of bulls.

[CR2] Amann RP, Wise WE, Glass JD, Nett TM (1986). Prepubertal changes in the hypothalamic pituitary in Holstein bulls. Biol Reprod.

[CR3] Bharath Kumar BS, Pandita S, Prakash BS, Mallick S, Mohanty TK, Mandal DK, Mili B (2015). Luteinizing hormone, testosterone and total estrogens response to exogenous GnRH in crossbred bulls with differing semen quality. Liv Sci.

[CR4] Bhavsar BK (1993). Perspective and prospective of the role of artificial insemination and gynecology in enhancing livestock production. Indian J Anim Reprod.

[CR5] El-Khawaga ARM, Kandiel MMM, Sosa GA, Abou El- Roos MEA, Abdel-Ghaffar AE, El Azab AESI (2011). Effect of GnRH analogue on libido and semen characteristics of puberal buffalo bulls. Benha Vet Med J Spec Issue.

[CR6] Gulia S, Sarkar M, Kumar V, Meyer HHD, Prakash BS (2010). Divergent development of testosterone secretion in male zebu (*Bos indicus*) and crossbred (*Bos indicus* × *Bos taurus*) and buffaloes (*Bubalus bubalis*) during growth. Trop Anim Health Prod.

[CR7] Horikawa R, Asakawa K, Hizuka N, Takano K, Shizume K (1989). Growth hormone and insulin-like growth factor I stimulate Leydig cell steroidogenesis. Eur J Pharmacol.

[CR8] Kearl LC (1982). Nutrient requirement of ruminant in developing countries.

[CR9] Kerr JB, Millar M, Maddocks S, Sharpe RM (1993). Stage-dependent changes in spermatogenesis and sertoli cells in relation to the onset of spermatogenic failure following withdrawal of testosterone. Anat Rec.

[CR10] Lin T, Haskell J, Vinson N, Terracio L (1986). Direct stimulatory effects of insulin-like growth factor-I on Leydig cell steroidogenesis in primary culture. Biochem Biophy Res Commun.

[CR11] Mallick S, Bharath Kumar BS, Prakash BS, Aggrawal A, Pandita S (2015). Development and validation of a simple, sensitive enzyme immunoassay for quantification of androstenedione in bull plasma. J Anim Sci Technol.

[CR12] Mc Lachlan RI, Wreford NG, Tsonis CKDM, Robertson DM (1994). Testosterone effects on spermatogenesis in the gonadotrophin-releasing hormone-immunized rat. Biol Reprod.

[CR13] Mondal M, Rajkhowa C, Prakash BS (2006). Relationship of plasma estradiol-17 β, total estrogen and progesterone to estrous behaviour in mithun (*Bos frontalis*) cows. Horm Behav.

[CR14] Mongkonpunya K, Hafs HD, Convey EM, Tucker HA, Oxender WD (1975). Serum luteinizing hormone, testosterone and androstenedione in pubertal and prepubertal bulls after gonadotropin releasing hormone. J Anim Sci.

[CR15] Mukhopadhyay CS, Gupta AK, Yadav BR, Khate K, Raina VS, Mohanty TK, Dubey PP (2010). Subfertility in males: an important cause of bull disposal in bovines. Asian Australas J Anim Sci.

[CR16] Pathirana IN, Kawate N, Büllesbach EE, Takahashi M, Hatoya S, Inaba T, Tamada H (2012). Insulin-like peptide 3 stimulates testosterone secretion in mouse Leydig cells via cAMP pathway. Regul Peptides.

[CR17] Prakash BS, Paul V, Anandlaxmi N (2002). Development and validation of a simple sensitive second antibody format enzymeimmunoassay for LH determination in plasma. J Immunol Methods.

[CR18] Sahni KL, Mohan G (1998). Annual report of animal reproduction division.

[CR19] Schanbacher BD, Echternkamp SE (1978). Testicular steroid secretion in response to GnRH-mediated LH and FSH release in bulls. J Anim Sci.

[CR20] Sethi RK, Raina VS, Joshi BK, Gurnani M (1989). Multistage selection of crossbred males and effect of their age and body weight on semen quality and freezability. Indian J Anim Sci.

[CR21] Sharpe RM, Knobil E, Neill JD (1994). Regulation of spermatogenesis. The physiology of reproduction.

[CR22] Verde LS, Trenkle A (1987). Concentrations of hormones in plasma from cattle with different growth potentials. J Anim Sci.

